# Combining Measures to Characterize Subcellular Machinery

**DOI:** 10.1371/journal.pbio.0020193

**Published:** 2004-06-15

Understanding how the cell functions—or breaks down—implies an understanding of the assembly lines, transportation systems, and powerhouses that keep it running. Global approaches are needed to identify the numerous proteins essential to each cellular machine. But which techniques are best? Lars Steinmetz and colleagues applied and evaluated a variety of methods to define mitochondrial proteins, and report that sets of complementary approaches are needed to characterize a cellular subsystem.[Fig pbio-0020193-g001]

**Figure pbio-0020193-g001:**
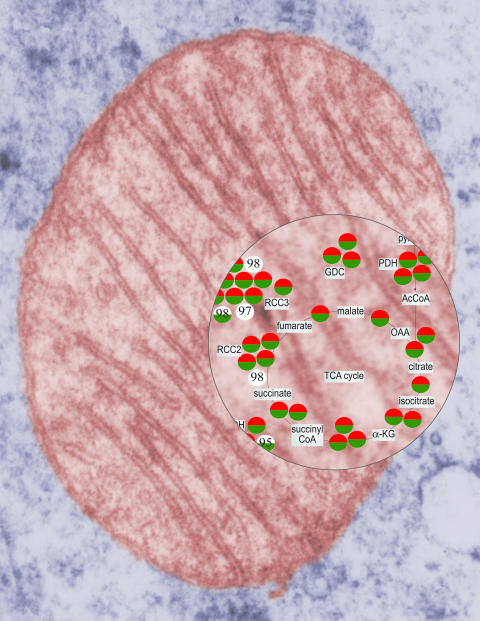
Zooming in on mitochondria (Image by Peter Seibel, design by Shayna Roosevelt)

Yeast mitochondria make an ideal subject for study. About two-thirds of the estimated 700 mitochondrial proteins have been identified to date, leaving fertile ground for new finds. The researchers previously compiled a list of 477 proteins with compelling evidence for mitochondrial involvement. This list provides a well-defined reference set against which to test protein-finding methods. Moreover, the well-studied and accessible yeast genome is well-suited for exploration and genetic manipulation. Since mitochondria are very similar among all eurkaryotes (organisms whose cells have nuclei), the results will prove relevant across species.

Steinmetz and colleagues triangulated results from multiple techniques to identify new candidate mitochondrial proteins. They compared the reference protein list to their new data from protein, mRNA, and gene knockout studies, and to 19 published datasets from other researchers, to evaluate the success of different techniques at finding known mitochondrial proteins. Then they combined evidence across studies to identify a set of proteins that likely characterizes most of the mitochondrial machinery.

The researchers first identified proteins from yeast mitochondria using a technique called liquid chromatography mass spectrometry, which separates the proteins by water insolubility (also called hydrophobicity), then identifies each by the mass and molecular charge of its constituents. By comparing this approach with others, the authors show that this proteomic technique alone is by no means comprehensive, nor error-free. Mass spectrometry is biased toward finding more abundant proteins, and the purified mitochondria can contain contaminants from elsewhere in the cell. To address these issues, the authors compared their protein data with a protein study from another group, the reference protein set, and a recent subcellular localization study. Potential mitochondrial proteins identified by more than one protein approach were more likely to localize to mitochondria in the localization study than were proteins identified by only one approach. This finding suggests that, compared to either method alone, a combination of protein and localization measures can more robustly identify proteins residing in mitochondria.

But since mitochondria, as the cell's power plants, are integrated into other cellular machinery, the authors argue, methods targeting proteins that are physically located to mitochondria should be complemented with functional approaches. Proteins with mitochondrial roles, regardless of concentration or location in the cell, are better identified by approaches that associate mRNA expression or gene deletion—which removes proteins or renders them inoperable—with changes in mitochondrial function.

By comparing results from multiple methods against the reference protein list, the researchers evaluated the likelihood that each protein was mitochondrial. They compiled a list of 691 top candidates. This multi-technique analysis easily outperformed any single study in terms of its ability to identify proteins in the reference set, and of the proportion of known versus unconfirmed proteins located. As mitochondria are well-conserved across species, the results provide a candidate gene list for finding human counterparts that might be associated with mitochondrial disorders.

Future studies can use this analysis to evaluate which research methods are likely to be most informative in other cell systems. This paper demonstrates the power of combining techniques with differing strengths in order to zero in on proteins that might elude any single approach, resulting in a more complete parts list for specific cellular machinery.

